# The allometry of proboscis length in Melittidae (Hymenoptera: Apoidae) and an estimate of their foraging distance using museum collections

**DOI:** 10.1371/journal.pone.0217839

**Published:** 2019-06-07

**Authors:** Annalie Melin, Harald W. Krenn, Rauri C. K. Bowie, Colin M. Beale, John C. Manning, Jonathan F. Colville

**Affiliations:** 1 Statistics in Ecology, Environment and Conservation, Department of Statistical Sciences, University of Cape Town, Cape Town, South Africa; 2 Compton Herbarium, South African National Biodiversity Institute, Claremont, South Africa; 3 Department of Integrative Zoology, University of Vienna, Faculty of Life Science, Vienna, Austria; 4 Department of Integrative Biology and Museum of Vertebrate Zoology, University of California—Berkeley, Berkeley, California, United States of America; 5 NRF Centre of Excellence at the Percy FitzPatrick Institute, University of Cape Town, Cape Town, South Africa; 6 Department of Biology, University of York, York, United Kingdom; 7 Research Centre for Plant Growth and Development, School of Life Sciences, University of KwaZulu-Natal, Pietermaritzburg, Scottsville, South Africa; 8 Kirstenbosch Research Centre, South African National Biodiversity Institute, Claremont, Cape Town, South Africa; University of California San Diego, UNITED STATES

## Abstract

An appreciation of body size allometry is central for understanding insect pollination ecology. A recent model utilises allometric coefficients for five of the seven extant bee families (Apoidea: Anthophila) to include crucial but difficult-to-measure traits, such as proboscis length, in ecological and evolutionary studies. Melittidae were not included although they are important pollinators in South Africa where they comprise an especially rich and morphologically diverse fauna. We measured intertegular distance (correlated with body size) and proboscis length of 179 specimens of 11 species from three genera of Melittidae. With the inclusion of Melittidae, we tested the between family differences in the allometric scaling coefficients. AIC model selection was used to establish which factors provide the best estimate of proboscis length. We explored a hypothesis that has been proposed in the literature, but which has not been tested, whereby body and range sizes of bees are correlated with rainfall regions. We tested this by using body size measurements of 2109 museum specimens from 56 species of Melittidae and applied the model coefficients to estimate proboscis length and foraging distance. Our results from testing differences across bee families show that with the addition of Melittidae, we retained the overall pattern of significant differences in the scaling coefficient among Apoidea, with our model explaining 98% of the variance in species-level means for proboscis length. When testing the relationship between body size and rainfall region we found no relationship for South African Melittidae. Overall, this study has added allometric scaling coefficients for an important bee family and shown the applicability of using these coefficients when linked with museum specimens to test ecological hypothesis.

## Introduction

Bees play a key functional role in almost all terrestrial ecosystems in pollinating both wild flowering plants [1–3] and agricultural crops [4,5]. Bees have also been identified as a potent co-evolutionary force in the diversification of plants [6–10] and in promoting gene flow (pollen transfer) among plant populations [11]. Key to these crucial ecological interactions is an understanding of their feeding preferences as determined in part by proboscis length and foraging distance, both of which have an allometric relationship with body size [12,13].

Proboscis length is an important ecological and evolutionary trait which influences bee flower choice [14–16] and foraging behaviour (e.g. flower handling) [17–21]. Functionally, the ability to take up nectar from a flower is dependent on the length of the labio-maxillary complex of the mouthparts. This functional unit comprises the main part of the proboscis and its length is crucial for nectar uptake from variously deep flowers [22]. The functional length of a bee’s proboscis is determined by both the distal glossa and the elongated prementum, which contains the musculature needed to move the glossa. When the labio-maxillary complex is fully extended for nectar uptake the glossa and prementum are more or less aligned and the sum of both lengths determines the functional length of the proboscis and the depth of the flowers from which the bee can access nectar [20,22].

Two principle proboscis morphologies can be distinguished in Apoidea. In “short-tongued” bees (i.e. Andrenidae, Colletidae, Melittidae and Halictidae) the glossa is shorter that the prementum whereas in “long-tongued” bees (i.e. Megachilidae and Apidae) (classification after Michener [23]) the glossa is longer than the prementum and a distinct food canal is formed by the elongated galeae and labial palpi that together surround the central glossa.

Cariveau et al. [12] highlight several challenges in measuring proboscis length of individual bee specimens, particularly small, short-tongued bees, in which the proboscis is flexed back under the head. To overcome these difficulties, they developed a predictive allometric equation to estimate proboscis length for five of the seven extant bee families, using easy-to-measure traits (e.g. body size) and taxonomic information (family). This approach has enabled proboscis length to be incorporated more readily into ecological studies [24–26] and its applicability has been extended to other bee families—most studies examining proboscis length have focused on the large-bodied *Bombus* (Apidae) [27–29].

Body size has also been found to be strongly correlated with foraging distance [13,30], and intertegular distance is a strong predictor of dry body mass [31,32]. Foraging range has been directly measured or estimated in only a few bee species [13,33–35]. Greenleaf et al. [13] developed a method using a power function to predict the relationship between body size and foraging distance. Cariveau et al. [12] incorporated Greenleaf et al.’s [13] equation into their method to estimate different categories of foraging distance, using it to calculate both proboscis length and foraging distance for five of the seven extant bee families. This expands our ability to investigate such aspects as bee foraging behaviour [9,13], resource competition [36], trait matching of pollinators with crops [37], and the structure of plant-pollinator networks [10].

Although Cariveau et al. [12] developed their method for application to the major bee families, they were unable to assess the families Melittidae (and see [32]), with only one common species in North America, and Stenotritidae, which are confined to Australia [38]. This is a significant limitation in regions in which Melittidae and Stenotritidae are important elements of the local bee fauna. Melittidae are widely distributed but absent from Australia and South America [23,39], with body size varying between 4 and 22 mm [40–42]. South Africa is a centre of diversity for Melittidae [42], with over 60 species recorded [43], some species representing among the earliest diversifying of all extant bee lineages [44], and the family has been the focus of several important pollination studies (e.g. [45–47]) and include a number of host-plant specialists [46,48] including morphological adaptations of forelegs for oil collection [49,50]. Understanding the foraging distance and flower preferences of these bees would complement these studies and add provide new insight.

Here, as part of a broader study assessing the patterns of functional diversity among South African bees, we apply to Melittidae the method for estimating foraging distance from body size measurements developed by Cariveau et al. [12] based on previously published equations [13,31]. We demonstrate the applicability of the model coefficients on new morphological data of South African Melittidae obtained from museum collections, by testing an intriguing hypothesis put forward by Kuhlmann in [51] and expanded in [52–54]. The author(s) propose that unfavourable climatic conditions (cold, windy, rainy) in the winter-rainfall region of South Africa restricts the daily foraging activity of the bees and thereby results in the small body size of bees emerging in winter and spring when floral resources are more abundant and diverse. It has been suggested that this selection on body size is due to small bee species being able to carry bigger pollen loads relative to their body size (i.e. increased foraging efficiency), than larger species. Given that the winter-rainfall region has high bee species richness [55], this hypothesis has also been used to explain bee alpha diversity by suggesting that the rate of speciation is increased due to a reduction in gene flow across the landscape as a consequence of the short flight ranges of the smaller bees and thereby the promotion of reproductive isolation among lineages.

It is well known that climatic conditions (e.g. temperature, wind speed, luminosity) affect the activity and flight of bees [56–59]. Contrary to the above hypothesis, generally larger bodied bees are considered better equipped to withstand cold and wet periods due to the well-developed ability to thermoregulate (pre-flight vibration of the wing muscles), in which there is an adjustment in body temperature in response to a wide range of climatic conditions [57,59,60]. Therefore, large bodied bees are partially able to overcome unfavourable climatic conditions when foraging for suitable resources [61]. When climatic conditions are unfavourable, smaller bees tend to start foraging later compared to larger bees, due to their limited ability to thermoregulate [57,61,62].

We test this hypothesis by assessing if bee size differs between rainfall seasonality regions. South African comprises distinct rainfall regions defined by rainfall seasonality [63] and this seasonality has been used to differentiate biogeographic areas including for bees [55,64].

## Material and methods

### Data collection and morphological measurements

To measure proboscis length on a range of species within the bee family Melittidae, we sampled 10 sites in the winter and summer rainfall areas of South Africa from September 2015 to March 2018 (S1 Table). Specimens collected were identified using the most recent keys [43,50,65,66] along with expert help. Voucher specimens have been deposited in the Iziko Museums of South Africa. Dissection of all proboscis were done on fresh specimens, using a Zeiss Stemi 305 stereo microscope. To measure the proboscis length and body size we used Leica Application Suite software (Ver. 4.7.1) on a Leica Z16 APO stereoscope.

**Fig 1:**
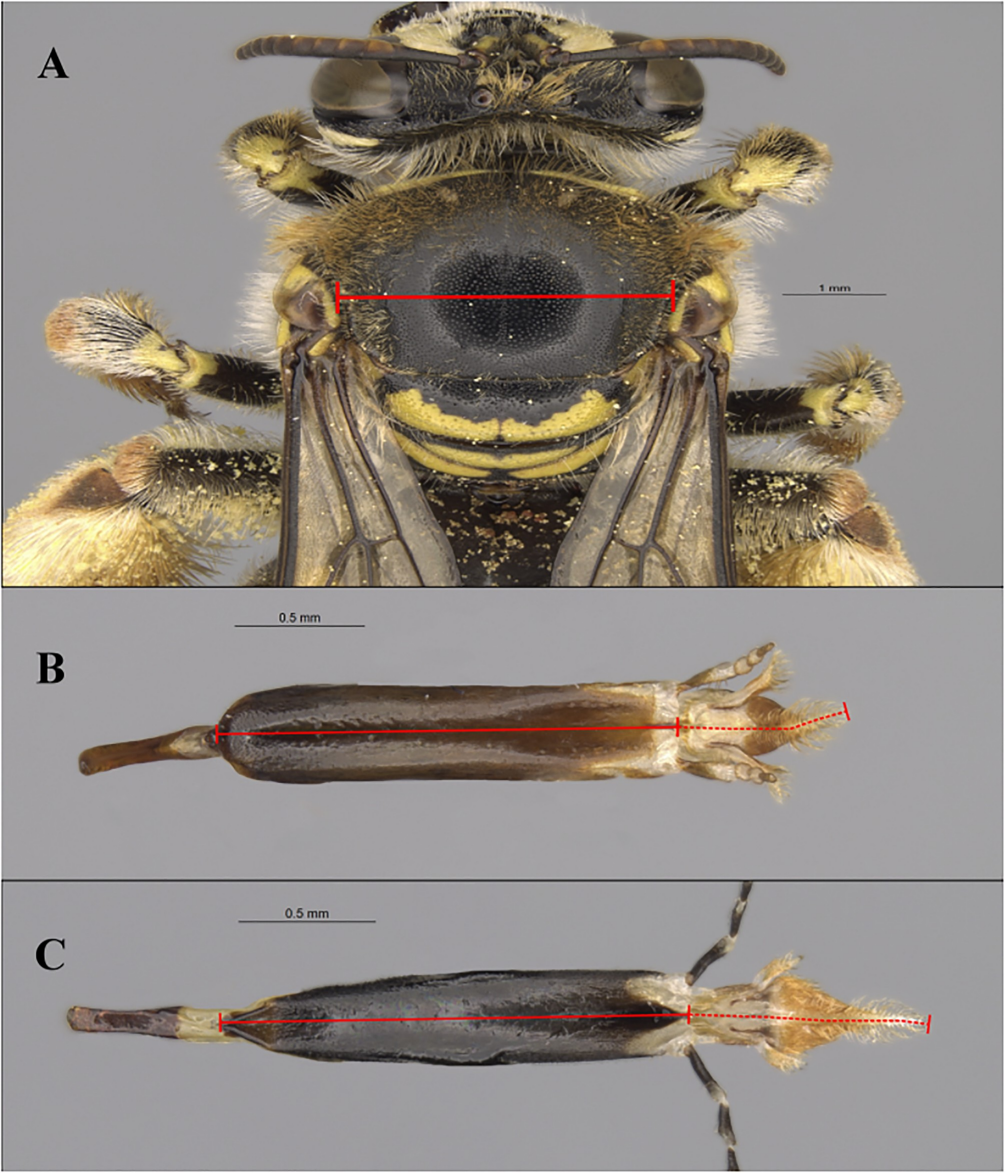
Photographs of the intertegular distance (IT) and dissected proboscis (glossa + prementum). **(A)** Photograph of IT for *Meganomia binghami* Cockerell (Melittidae) ♀**(B)** Glossa (depicted by dashed red lines) and prementum (depicted by solid red lines) length for short-tongued Melittidae bees *Meganomia binghami* ♀ and **(C)** *Redivivoides simulans* Michener ♀.

Here we employed the same definitions and techniques to measure proboscis length and body size as in Cariveau et al. [12] for reasons of comparability. We summarise these as follows. Proboscis length is the combined length of the glossa and prementum. The prementum was measured from the proximal base of the mentum to the tip of the basioglossal sclerite [23] (Fig 1B and 1C). The length of the glossa was taken from the basiglossal sclerite to the distal end of the labellum [67]. We took all measurements only when the glossae was fully extended from the prementum [67]. We measured the intertegular distance (IT), a standard measure of body size [31,68,69]), between the tegulae at the wing bases (Fig 1A).

To estimate proboscis length and foraging distance for Melittidae, we measured the IT of pinned specimens identified to species obtained from the three main bee collections in South Africa: Iziko Museums of South Africa, Albany Museum, and the National Collection of Insects [70]. We measured between 1 and 176 specimens per species (when the sex of the specimen has been included on the determination label we endeavoured to measure both males and females) across seven genera were measured depending on availability. If we encountered a long series of specimens collected at the same place and same time, we measured the first ten in the unit tray [71].

### Data analysis

The purpose of this paper is to build-on the existing work by Cariveau et al. [12], we therefore followed their data analysis protocol by using an allometric power function to test the interspecific relationship between proboscis length, taxonomy (family) and intertegular distance. To allow for direct comparison of results, we combined our dataset for Melittidae with the authors ([12]; S2 Table) dataset. We fitted OLS regression models with overall proboscis length as the response variable with separate models run for prementum and glossa lengths, because these measurements may also be functionally important for the type of flowers visited [18,67]. Both the response and explanatory variables were log-transformed [12].

In contrast to Cariveau et al’s analyses, the majority of individuals collected in our sample were composed of males. The OLS regression models were therefore run with only males versus with both females and males. Because there were small differences between males and male and female models (S2 and S3 Tables), we fitted regression models to test whether the slope of the relationship between IT and mouthpart differed between both sexes and species, or only by sex. These models showed that both males and females have the same slope (S4 Table) and therefore we also combined the sexes in further analyses.

We used the lowest Akaike Information Criterion (AIC) value to select which variables provided for the best supported model [72]. As with Cariveau et al., should the best-fitting model include the family coefficient it suggests that the intercepts differ between families. If the best-fitting model includes the scaling coefficient it suggests that proboscis, glossa or prementum scales with IT. The presence of an interaction between family and IT suggests that the scaling coefficient differs between families. Additional model selection was performed to ascertain if tongue-type (long- or short-tongued) alone predicts proboscis length and if tongue-type, IT and their interaction improves model fit. The model with only tongue-type does not account for allometry. We parameterized the allometric power function using the estimate values from the best fitting models.

Finally, we used the mean IT for each species of Melittidae obtained from museum specimens to calculate the proboscis, prementum and glossa length and typical and maximum foraging distance. We incorporated the family coefficients for Melittidae from the OLS regression models as in Cariveau et al. [12] based on previously published equations [13,31]. Using the distributional data from the measured specimens and digitised specimens from the three main bee collections in South Africa, we overlaid these georeferenced points with simplified rainfall regions (winter, aseasonal, early summer, and late summer; S1 Fig) based on rainfall seasonality [63] following [55] to determine the number of species in each region (S1 Fig). We summarise species trait data: body size (IT), proboscis (gloss + prementum) length and foraging distance in terms of the four rainfall regions. The number of species per rainfall region are as follows: winter = 44; aseasonal = 13; early summer = 12; and late summer = 20).

To test if species body size is significantly different between rainfall regions and to control for phylogenetic non-independence [73–76], we employed two approaches. For the first approach, we reconstructed and updated the phylogeny for 77 species of Melittidae ([77]; S6 Table, S1 Methods & Results, S2 and S3 Figs) and used it as a backbone to construct an applicable phylogeny for our trait dataset. We pruned the phylogeny to only those genera that occur in South Africa (S6 Table) and added species tips to genera nodes as polytomies of equal branch-length relative to the genera branch-length (similar in approach to [32]). We excluded five South Africa species (*Afrodasypoda plumipes*, *Capicola hantamensis*, *Melitta avontuurensis*, *Melitta richtersveldensis*, *Samba spinosa*) from the updated phylogeny because we did not have trait data for these species. We make the assumption that most variation in body size occurs at and above the genus level; however, this is not completely unwarranted [32]. We then fitted a phylogenetic generalized least-squares (PGLS) linear model to the trees, with IT (log-transformed) as the response variable, rainfall region as the explanatory variable, and with a Brownian motion error structure. We used ANOVA to test the effect of rainfall region of body size against a null model.

Because our phylogenetic analysis only contained meaningful branch length information at genus and above, we complemented this analysis with a second method, using taxonomy (genus, tribe and subfamily) to account for evolutionary history [32,78]. To this data, we fitted linear mixed-effects models (LMM), with IT (log-transformed) as the response variable and rainfall region as the explanatory variable. We considered taxonomy as a nested random effect and performed a maximum likelihood test and used AIC against a null model to select for the best supported model.

All analyses were carried out using the software R [79], using packages “nlme” [80] to run the linear models and “lme4” [81] to run the LMMs, “ape”[82] to prune the phylogeny. To produce the accompanying figures we used the package “ggplot2” [83].

## Results

We measured 179 specimens from 11 species belonging to the three genera *Meganomia*, *Rediviva* and *Redivivoides* (Melittidae). Melittidae are well-represented in South Africa, with 67 species in eight genera [43] and we were able to obtain a representative sample for the region (~ 40% of genera). We provide the mean IT, glossa, prementum and proboscis for each species in supplementary information (S5 Table).

The inclusion of Melittidae in the OLS regression provides a better fit (Cariveau et al. [12], Table 1) for the prementum (R^2^ = 0.93) and proboscis models (R^2^ = 0.98) but not for the glossa model (R^2^ = 0.90). The best-fitting models, based on AIC scores (Table 1), include both family and IT, which strongly predicts the mean length of proboscis, glossa and prementum. The best-fitting models for proboscis and glossa were additive whereas the prementum model was improved by an interaction between family and IT (Table 1).

**Table 1:** Summary of model selection statistics for interspecific OLS regression models. Models are listed in order of increasing AIC value with the best model (lowest AIC) depicted in bold.

Response variable	Model	Adjusted R^2^	AIC
**Proboscis**	**Family + IT**	**0.98**	**-52.62**
	Family × IT	0.98	-50.40
	Short- vs. Long-Tongued + IT	0.97	-22.68
	Short- vs. Long-Tongued × IT	0.97	-23.05
	IT Only	0.92	83.45
	Family Only	0.91	103.65
	Short- vs. Long-Tongued Only	0.90	105.84
**Glossa**	**Family + IT**	**0.90**	**66.48**
	Family × IT	0.90	70.13
	Short- vs. Long-Tongued × IT	0.87	99.23
	Short- vs. Long-Tongued + IT	0.86	103.10
	Family Only	0.78	156.48
	Short- vs. Long-Tongued Only	0.77	160.13
	IT Only	0.22	296.72
**Prementum**	**Family** × **IT**	**0.93**	**-114.02**
	Family + IT	0.92	-105.32
	Short- vs. Long-Tongued × IT	0.90	-71.18
	Short- vs. Long-Tongued + IT	0.89	-71.19
	IT Only	0.87	-50.23
	Family Only	0.66	64.18
	Short- vs. Long-Tongued Only	0.61	77.17

**Fig 2:**
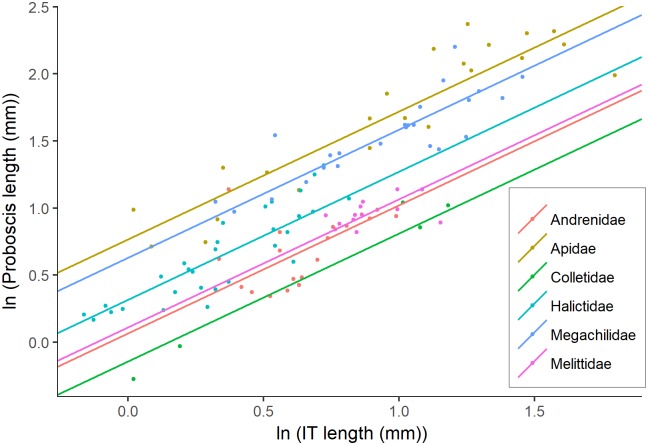
Allometric relationship between IT and proboscis length. The relationship between intergular distance (IT) and proboscis length in 11 species from Melittidae and 100 species (Cariveau et al. [12], S2 Table) from Apidae, Megachilidae, Andrenidae, Collectidae and Halictidae. The mean IT and proboscis length for each species is depicted as a point. Each bee family is represented by a colour. The fitted lines are based on regression coefficients from model outputs. Proboscis length and IT are both ln transformed.

**Fig 3:**
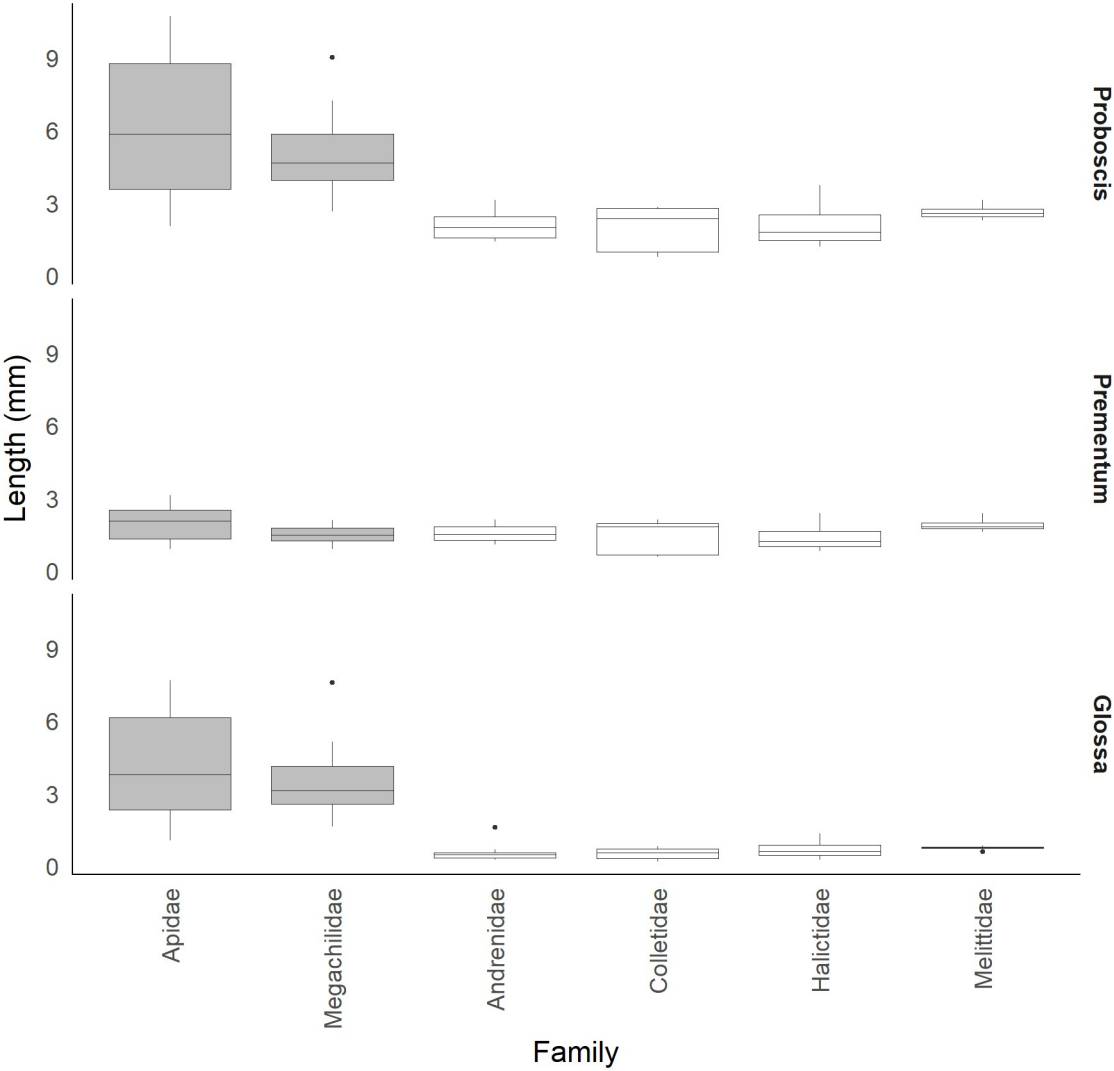
Length of bee mouthparts (proboscis, glossa and prementum). Boxplots of proboscis, glossa and prementum length for six bee families. Long-tongued families are depicted by grey boxplots whereas short-tongued families are depicted as white boxplots. Outliers are shown as dots. Figures are drawn using raw data for Melittidae and data from (Cariveau et al. [12], S2 Table) for Apidae, Megachilidae, Andrenidae, Collectidae and Halictidae.

In all best-fitting models (Table 1), the grouping variable family was retained with the inclusion of Melittidae, strengthening the finding that the mean lengths of the glossa and prementum differed among families (Fig 2). With the addition of the short-tongued family Melittidae, the overall pattern was retained, whereby proboscis and glossa lengths differed among long- and short-tongued families, and prementum length was more similar (Table 2, Fig 3). The addition of Melittidae also resulted in low R^2^ values for models fit with family only (glossa = 0.78, prementum = 0.66, proboscis = 0.91, Table 1) or only with long vs. short-tongued family groups (glossa = 0.77, prementum = 0.61, proboscis = 0.90, Table 1), or IT only (glossa = 0.22, prementum = 0.87, proboscis = 0.92, Table 1).

**Table 2:** The parameter values for the allometric power function [12] using the estimates from the best fitting (lowest AIC) OLS regression models (Table 1). Logs are in base e.

Response variable	Family	Family-specific coefficient	IT scaling coefficient
**Proboscis**	Andrenidae	1.06	
	Apidae	2.15	
	Colletidae	0.86	
	Halictidae	1.37	
	Megachilidae	1.87	
	Melittidae	1.10	
		—	0.96
**Prementum**	Andrenidae	0.88	0.83
	Apidae	0.91	0.75
	Colletidae	0.56	1.14
	Halictidae	0.89	1.05
	Megachilidae	0.76	0.70
	Melittidae	1.26	0.45
**Glossa**	Andrenidae	0.23	
	Apidae	1.28	
	Colletidae	0.21	
	Halictidae	0.42	
	Megachilidae	1.17	
	Melittidae	0.29	
		—	1.04

We parameterized the allometric power function given in Cariveau et al. ([12], Eq1) using the estimates from the best-fitting models that include Melittidae. The best-fitting model for the proboscis and glossa does not include an interaction term between family and IT (Table 1). This indicates that the slopes do not differ across families and therefore the value for the IT scaling coefficient is the same for each family. Whereas the best-fitting prementum model includes an interaction term between IT and family, the IT scaling coefficient therefore differs for each family. We provide a summary table of the model-estimated values for the family-specific coefficients and IT scaling coefficients in Table 2. Addition of Melittidae to the sampling does not affect the finding that the allometric scaling relationship between IT and proboscis length still differs among families. The relationship between IT and proboscis length is linear based on the IT scaling coefficient being close to 1 (Table 2).

### Estimating proboscis length and foraging range of Melittidae

**Fig 4:**
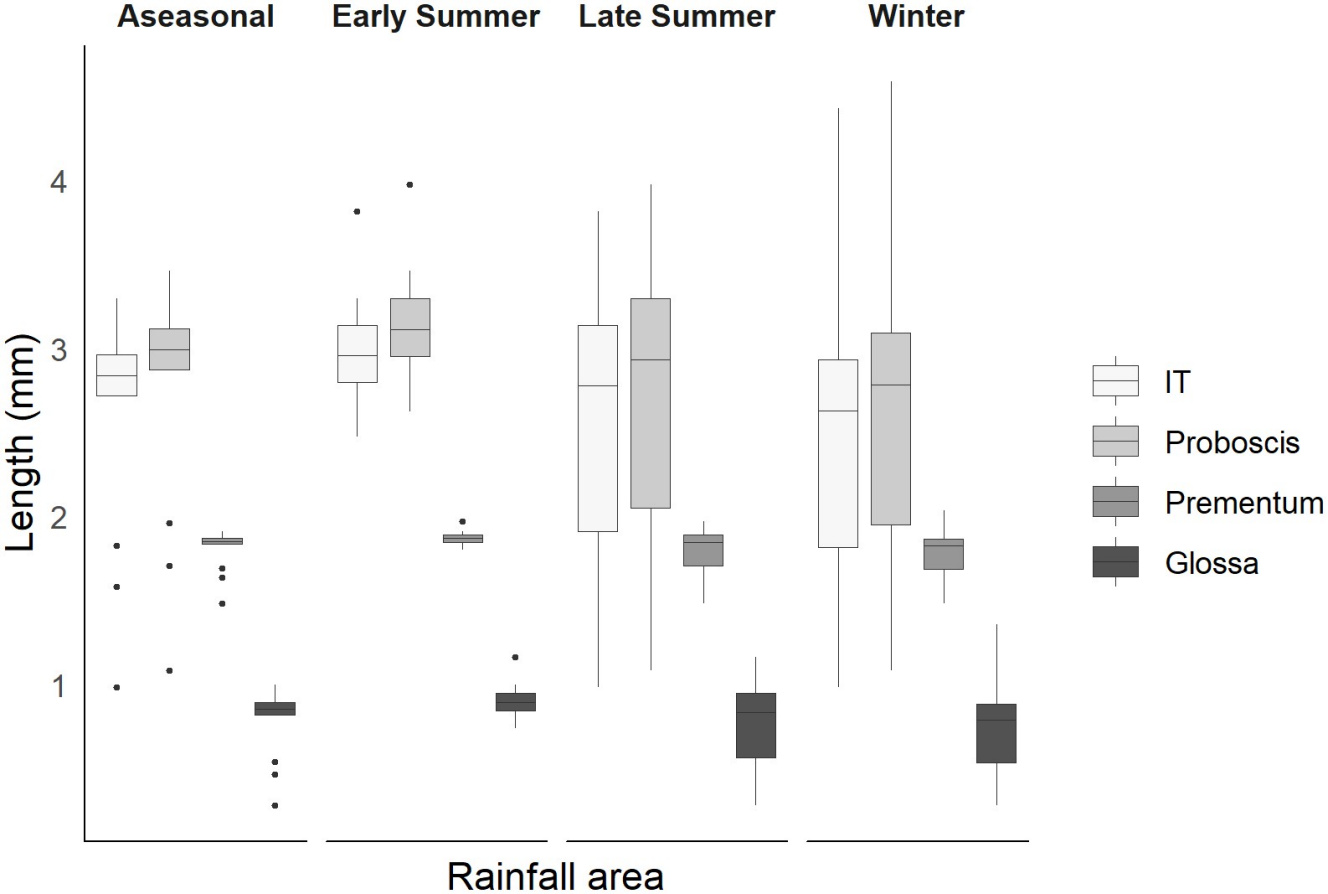
Estimated length of mouthparts (proboscis, prementum and glossa) across rainfall regions. Each panel represents the four rainfall regions: aseasonal, early summer, later summer and winter. Boxplots of measured IT and estimated proboscis, prementum and glossa length for 56 species of Melittidae. Dots represent outliers. Proboscis, prememtum and glossa length was estimated from measured IT using family-specific scaling coefficients.

**Fig 5:**
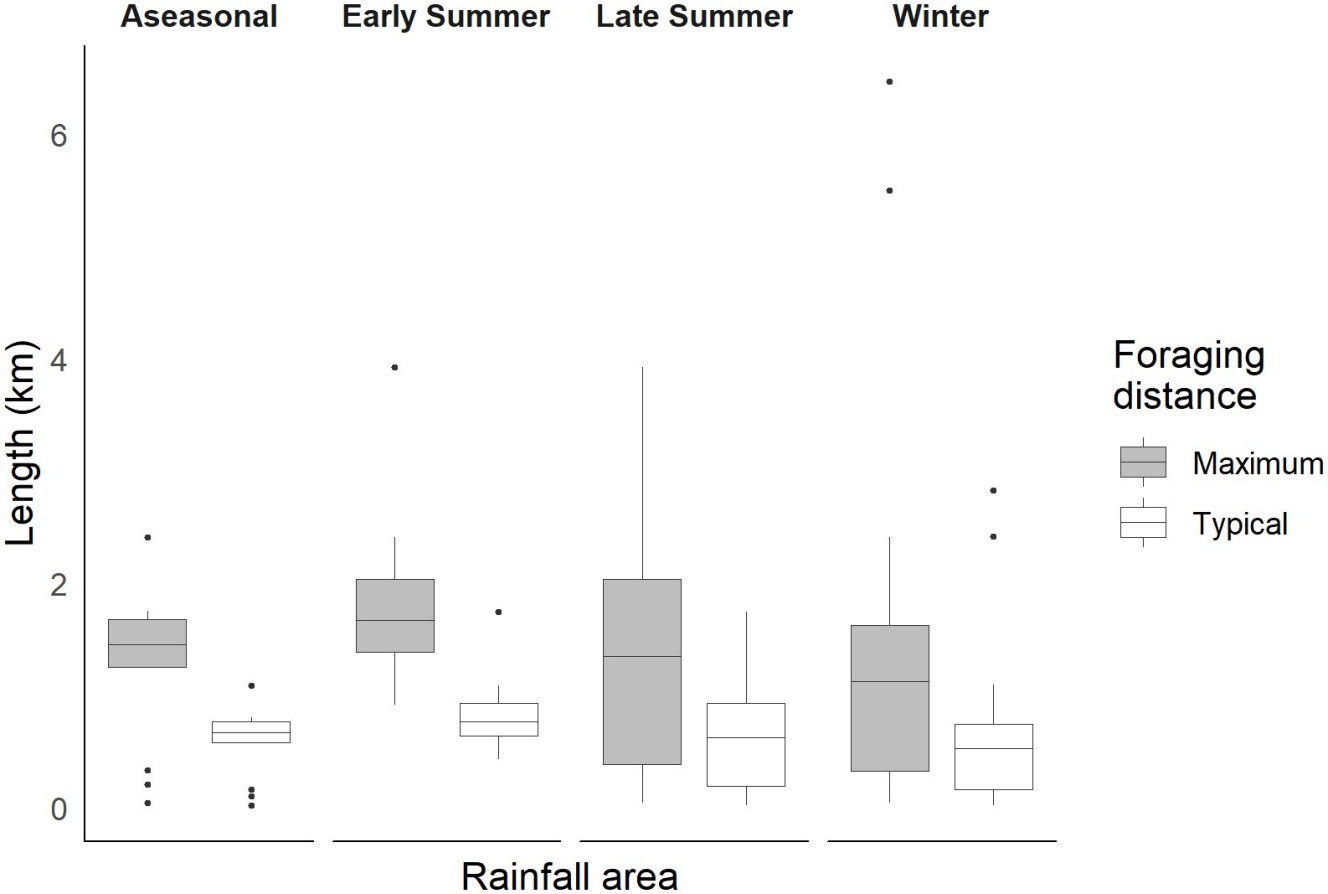
Estimated typical and maximum bee foraging distance across rainfall regions. Each panel represents the four rainfall regions: aseasonal, early summer, later summer and winter. Boxplots of typical and maximum foraging distance for 56 species of Melittidae. Dots represent outliers. Typical and maximum foraging distance were estimated form IT using previously published equations [13].

We measured 2109 specimens from 56 species belonging to seven genera, representing 89% of the Melittidae species in South Africa. Using the mean IT for each species we estimated the proboscis, glossa and prementum length and the typical and maximum foraging distance for each of the four rainfall regions in the subcontinent distinguished by rainfall seasonality (Figs 4 and 5). Most species (~55%) were restricted to a single rainfall region, with only ~32% overlapping across two regions, and ~2% overlapping across all four regions. Body size (IT) for Melittidae species ranges between 0.99 and 4.42 mm (mean = 2.58 mm). We found no significant relationship between rainfall region and mean body size when controlling for phylogeny (best parsimony tree: *F*_(1, 3)_ = 0.10, p = 0.98; maximum likelihood tree: *F*_(1, 3)_ = 0.05, p = 0.99). Similarly, when using taxonomy, we found that rainfall region had no effect on mean body size; the null model was the best-fitting model based on the AIC score (S8 Table), with all rainfall regions showing bees with similar sized IT (Fig 4).

## Discussion

This study established the body size allometric scaling relationship for the bee family Melittidae, thereby expanding the work by Cariveau et al. [12] to include six of the seven extant bee families. We then applied this family-specific allometric equation, with an estimation for the scaling coefficient between IT and proboscis length, to body size measurements obtained from museum specimens to estimate the proboscis length and foraging distance for Melittidae. In addition, using body size measurements we estimated foraging distance following Greenleaf et al. [13]. Using this trait information for South African species of Melittidae, we were able to examine body size, proboscis length and foraging range patterns in relation to rainfall seasonality.

Cariveau et al. [12] identified significant differences across bee families in their scaling coefficients. The inclusion of Melittidae did not change this pattern but slightly improved the variance (98%) associated with species-level means for proboscis length across families. The allometric body size scaling relationship remains to be established for the endemic Australian Stenotritidae. This family is closely allied to Colletidae, and it remains to be determined whether it will have the same coefficient as that family or not [44,84,85].

In order to make their results accessible, Cariveau et al. [12] created an R package *BeeIT* [86] which was recently reimplemented by Kendall et al. [32] in package *Pollimetry* [87], which are simple to use, requiring only the input of family information and body size measurements to obtain an estimate of proboscis length. We are able to contribute to these packages by providing the scaling coefficients for Melittidae, allowing for the estimation of proboscis length for species of this family, an important component of the bee fauna in some regions. This will complement many of the ecological and evolutionary studies in Melittidae, such as host-plant associations and foraging range [48,88], competition [89], shifts in species range and host-plants [90], co-evolution with host-plants [45–47,91–95], morphological adaptations for oil-collecting [96], and location of nest sites [97,98]. Although there has been a particular focus on the oil-collecting bees *Rediviva* and the co-evolution of their host plants [45,46,99], their functional traits, including foraging range and behaviour (e.g. proboscis length), have not been considered in these studies, nor for any other South African bee species. As we are currently investigating patterns of foraging distance and behaviour at a regional scale for South African bees, having the scaling coefficients for all six families that are regionally represented is an important step for our analyses and for future studies that may require such data.

We demonstrate one component of this, by applying the allometric scaling coefficient for Melittidae to measured museum specimens to estimate foraging distance and proboscis length in order to relate these traits to an environmental variable (rainfall-seasonality) of putative importance for bee diversity [55,100]. It has been hypothesised that rainfall-seasonality is correlated with body size of bees and that this has important implications for their co-evolutionary relationships and patterns of speciation with host plants [52]. Our data, which incorporated ~90% of South African Melittidae species, suggest that there is no apparent relationship between rainfall-seasonality and bee body size even when controlling for evolutionary history (see also [32]). Determinants of insect body size are complicated but general predictions suggest that body size will correlate with temperature and larval resource availability, and that relatively larger-sized bees should be more common in the cool season rainfall areas of South Africa [101–103]. Although species of the summer-rainfall genus *Meganomia* (mean IT: ± SD 3.813 ± 0.267) are considered to be large-sized bees, species of winter-rainfall *Rediviva* (mean IT: ± SD 2.948 ± 0.371) and *Redivivoides* (mean IT: ± SD: 2.650 ± 0.348) are also of impressive size. In additional to the climate, body size in winter-rainfall Melittidae is possibly further influenced by the abundant spring floral resources available for larval nutrition and development, including nutrient-rich floral oils [50,104–106], allowing for larger-sized adults. Whether the pattern we have retrieved for Melittidae holds across other bee families remains to be tested. We are currently investigating correlates of bee body size across fine-scale environmental and plant diversity gradients for all six South African bee families and hope to be able to tease apart the role environmental and/or plant resources play in structuring bee diversity.

In conclusion, investigating allometric relationships in body size has been shown to be key for understanding components of species ecology and evolution [12,107,108]. The methodology developed by Cariveau et al. [12] allows this important trait data to be easily determined from specimens and therefore included in these kinds of studies. Our data could be incorporated into their tool and its reimplementation [32] by adding an important bee family. We have demonstrated its applicability when linked with museum specimens to test environmental correlates of bee body size and diversity.

## Supporting information

S1 Table Locality information for sampled sites in the winter and summer rainfall areas of South Africa. (XLSX)

S2 Table Summary of model selection statistics for interspecific OLS regression models of male only data. Models are listed in order of increasing AIC value with the best model (lowest AIC) depicted in bold.
(DOCX)

S3 Table The parameter values for the allometric power function [12] using the estimates from the best fitting (lowest AIC) OLS regression models (S2 Table) of male only data. Logs are in base e.
(DOCX)

S4 Table Summary statistics for regression models. Testing whether the slope of the relationship between IT and mouthpart (proboscis, prementum, glossa) differed between both sexes and species, or only by sex. Species (n = 11), Sex = Male or Female, IT = intergular length (mm), n.s. = not significant. Logs are in base e.
(DOCX)

S5 Table Table of species-level means for IT, glossa, prementum and proboscis length for Melittidae. (XLSX)

S6 Table Description of the molecular dataset used to build an updated Melittidae phylogeny. We used the data from Michez et al. [109] as a foundation and supplemented these data with available molecular data from GenBank primarily sourced from (Michez et al. 2010; Dellicour et al. 2014; Kahnt et al. 2017). Records in bold are species that occur in South Africa.
(XLSX)

S7 Table Combined DNA data matrix in both nexus and excel file formats. (ZIP)

S8 Table Summary of model selection statistics for the taxonomy-based interspecific OLS regression models. Testing whether rainfall regions has an effect on body size (IT) including taxonomy. IT = intergular length (mm), rainfall regions = winter, aseasonal, early summer, and late summer. Models are listed in order of increasing AIC value with the best model (lowest AIC) depicted in bold. Logs are in base e.
(DOCX)

S1 Fig Map of simplified rainfall regions in South Africa based on Shultze and Maharaj [63] and following Kuhlmann [55]. (PDF)

S2 Fig One of 48 equally parsimonious trees based on the combined analysis of eight genes. Numbers below nodes are bootstrap support values.
(PDF)

S3 Fig Maximum likelihood tree based on the combined analysis of eight genes. Numbers below nodes are bootstrap support values.
(PDF)

S1 Methods & Results Construction of an updated phylogenetic hypotheses of the Melittidae. (DOCX)
