# Supplementary material for: The allometry of proboscis length in Melittidae (Hymenoptera: Apoidae) and an estimate of their foraging distance using museum collections
Source: PLoS One. 2019 Jun 7;14(6):e0217839. doi: 10.1371/journal.pone.0217839 (PMC6555519; doi:10.1371/journal.pone.0217839)
Supplement: S2 Table — Models are listed in order of increasing AIC value with the best model (lowest AIC) depicted in bold. (DOCX) [file pone.0217839.s002.docx]

**S2 Table. Summary of model selection statistics for interspecific OLS regression models of male only data**. Models are listed in order of increasing AIC value with the best model (lowest AIC) depicted in bold.

| **Response variable** | **Model** | **R^2^** | **AIC** |
| --- | --- | --- | --- |
| **Proboscis** | **Family + IT** | **0.98** | **-47.02** |
|  | Family × IT | 0.98 | -45.87 |
|  | Short- vs. Long-Tongued × IT | 0.97 | 18.85 |
|  | Short- vs. Long-Tongued + IT | 0.97 | 18.34 |
|  | IT Only | 0.92 | 82.51 |
|  | Short- vs. Long-Tongued Only | 0.91 | 103.59 |
|  | Family Only | 0.91 | 103.97 |
| **Glossa** | **Family + IT** | **0.91** | **68.24** |
|  | Family × IT | 0.91 | 72.00 |
|  | Short- vs. Long-Tongued × IT | 0.87 | 99.89 |
|  | Short- vs. Long-Tongued + IT | 0.86 | 103.62 |
|  | Family Only | 0.79 | 155.68 |
|  | Short- vs. Long-Tongued Only | 0.77 | 157.99 |
|  | IT Only | 0.23 | 289.28 |
| **Prementum** | **Family** × **IT** | **0.94** | **-109.27** |
|  | Family + IT | 0.93 | -98.87 |
|  | Short- vs. Long-Tongued + IT | 0.90 | -66.06 |
|  | Short- vs. Long-Tongued × IT | 0.90 | -65.56 |
|  | IT Only | 0.87 | -47.91 |
|  | Family Only | 0.67 | 65.13 |
|  | Short- vs. Long-Tongued Only | 0.61 | 74.04 |
