# Supplementary material for: The allometry of proboscis length in Melittidae (Hymenoptera: Apoidae) and an estimate of their foraging distance using museum collections
Source: PLoS One. 2019 Jun 7;14(6):e0217839. doi: 10.1371/journal.pone.0217839 (PMC6555519; doi:10.1371/journal.pone.0217839)
Supplement: S3 Table — Logs are in base e. (DOCX) [file pone.0217839.s003.docx]

**S3 Table.** **The parameter values for the allometric power function (Cariveau et al. 2016)** **using the estimates from the best fitting (lowest AIC) OLS regression models (S1 Table) of male only data.** Logs are in base e.

| **Response variable** | **Family** | **Family-specific coefficient** | **IT scaling coefficient** |
| --- | --- | --- | --- |
| **Proboscis** | Andrenidae | 1.07 |  |
|  | Apidae | 2.15 |  |
|  | Colletidae | 0.86 |  |
|  | Halictidae | 1.37 |  |
|  | Megachilidae | 1.88 |  |
|  | Melittidae | 1.10 |  |
|  |  | --- | 0.95 |
| **Prementum** | Andrenidae | 0.88 | 0.83 |
|  | Apidae | 0.91 | 0.72 |
|  | Colletidae | 0.56 | 1.13 |
|  | Halictidae | 0.89 | 1.04 |
|  | Megachilidae | 0.76 | 0.67 |
|  | Melittidae | 1.49 | 0.21 |
| **Glossa** | Andrenidae | 0.23 |  |
|  | Apidae | 1.28 |  |
|  | Colletidae | 0.21 |  |
|  | Halictidae | 0.42 |  |
|  | Megachilidae | 1.17 |  |
|  | Melittidae | 0.29 |  |
|  |  | --- | 1.04 |
