# Supplementary material for: The allometry of proboscis length in Melittidae (Hymenoptera: Apoidae) and an estimate of their foraging distance using museum collections
Source: PLoS One. 2019 Jun 7;14(6):e0217839. doi: 10.1371/journal.pone.0217839 (PMC6555519; doi:10.1371/journal.pone.0217839)
Supplement: S4 Table — Testing whether the slope of the relationship between IT and mouthpart (proboscis, prementum, glossa) differed between both sexes and species, or only by sex. Species (n = 11), Sex = Male or Female, IT = intergular length (mm), n.s. = not significant. Logs are in base e. (DOCX) [file pone.0217839.s004.docx]

**S4 Table.** **Summary statistics for regression models.** Testing whether the slope of the relationship between IT and mouthpart (proboscis, prementum, glossa) differed between both sexes and species, or only by sex. Species (n = 11), Sex = Male or Female, IT = intergular length (mm), n.s. = not significant. Logs are in base e.

| **Model** | **Response variable** | **Explanatory variables** | **d.f.** | **F-value** | ***p*** |
| --- | --- | --- | --- | --- | --- |
| **Model 1** | ln(proboscis length) | Species | 5 | 66.45 | <0.001 |
|  |  | ln(IT) × Sex | 1 | 2.68 | n.s. |
| **Model 2** | ln(proboscis length) | ln(IT) × Species | 5 | 2.52 | 0.03 |
|  |  | ln(IT) × Sex | 1 | 2.92 | n.s. |
| **Model 3** | ln(prementum length) | Species | 5 | 88.69 | <0.001 |
|  |  | ln(IT) × Sex | 1 | 3.11 | n.s. |
| **Model 4** | ln(prementum length) | ln(IT) × Species | 5 | 2.91 | 0.02 |
|  |  | ln(IT) × Sex | 1 | 3.84 | n.s. |
| **Model 5** | ln(glossa length) | Species | 5 | 14.12 | <0.001 |
|  |  | ln(IT) × Sex | 1 | 0.42 | n.s. |
| **Model 6** | ln(glossa length) | ln(IT) × Species | 5 | 0.82 | n.s. |
|  |  | ln(IT) × Sex | 1 | 0.38 | n.s. |
