# Supplementary material for: The allometry of proboscis length in Melittidae (Hymenoptera: Apoidae) and an estimate of their foraging distance using museum collections
Source: PLoS One. 2019 Jun 7;14(6):e0217839. doi: 10.1371/journal.pone.0217839 (PMC6555519; doi:10.1371/journal.pone.0217839)
Supplement: S8 Table — Testing whether rainfall regions has an effect on body size (IT) including taxonomy. IT = intergular length (mm), rainfall regions = winter, aseasonal, early summer, and late summer. Models are listed in order of increasing AIC value with the best model (lowest AIC) depicted in bold. Logs are in base e. (DOCX) [file pone.0217839.s008.docx]

**S8 Table.** **Summary of model selection statistics for the taxonomy-based interspecific OLS regression models**. Testing whether rainfall regions has an effect on body size (IT) including taxonomy. IT = intergular length (mm), rainfall regions = winter, aseasonal, early summer, and late summer. Models are listed in order of increasing AIC value with the best model (lowest AIC) depicted in bold. Logs are in base e.

| **Model formula** | **d.f.** | **AIC** |
| --- | --- | --- |
| **ln(IT)~1 + (1\|subfamily/tribe/genus)** | **5** | **-36.11** |
| ln(IT) ~Rainfall + (1\|subfamily/tribe/genus) | 8 | -30.28 |
