# Supplementary figures and images for: The allometry of proboscis length in Melittidae (Hymenoptera: Apoidae) and an estimate of their foraging distance using museum collections
Source: PLoS One. 2019 Jun 7;14(6):e0217839. doi: 10.1371/journal.pone.0217839 (PMC6555519; doi:10.1371/journal.pone.0217839)

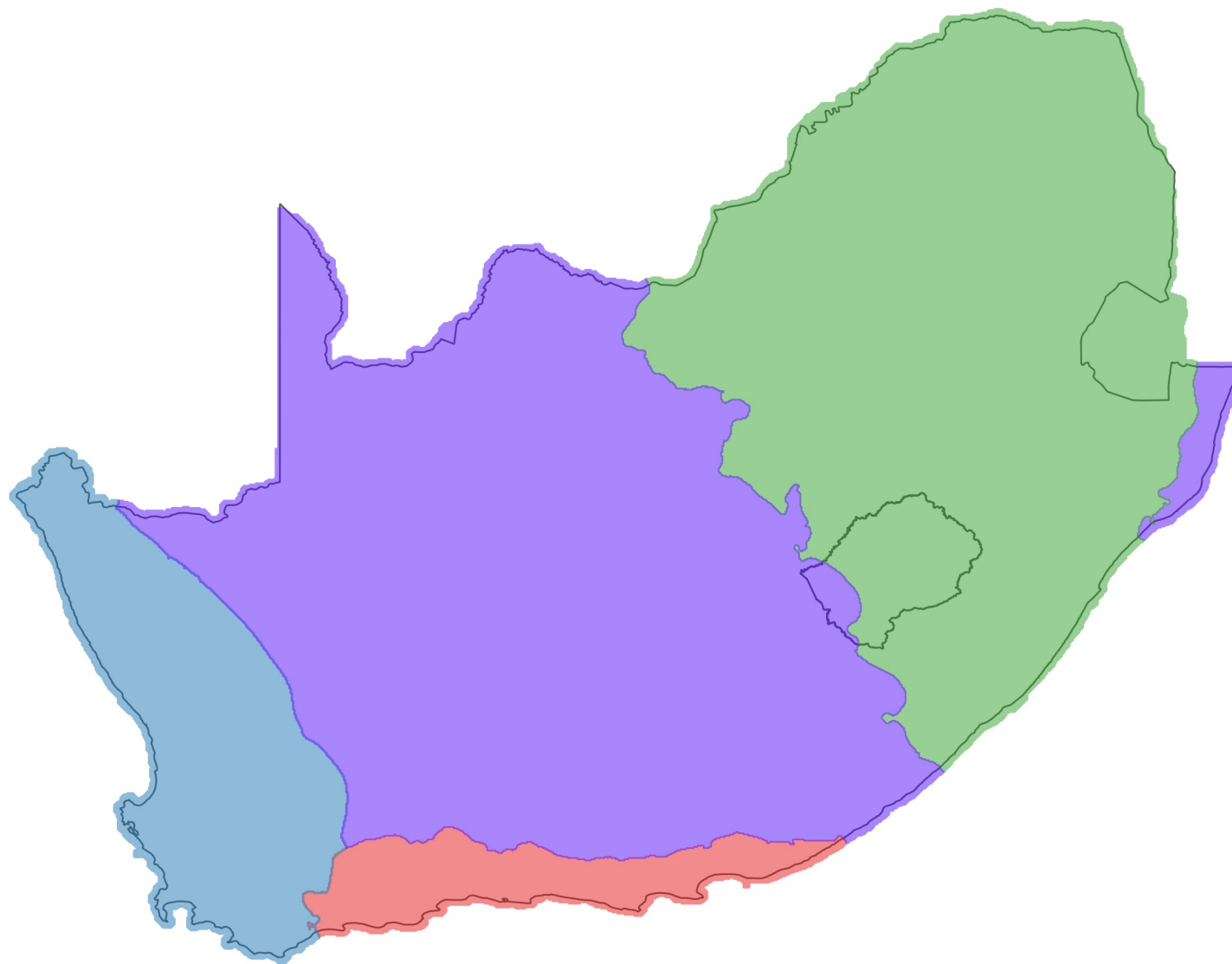

### Rainfall regions

- 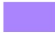 Late summer
- 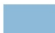 Winter
- 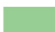 Early summer
- 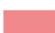 Aseasonal
- 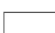 South Africa

Supplement: S1 Fig — (PDF) [file pone.0217839.s009.pdf]

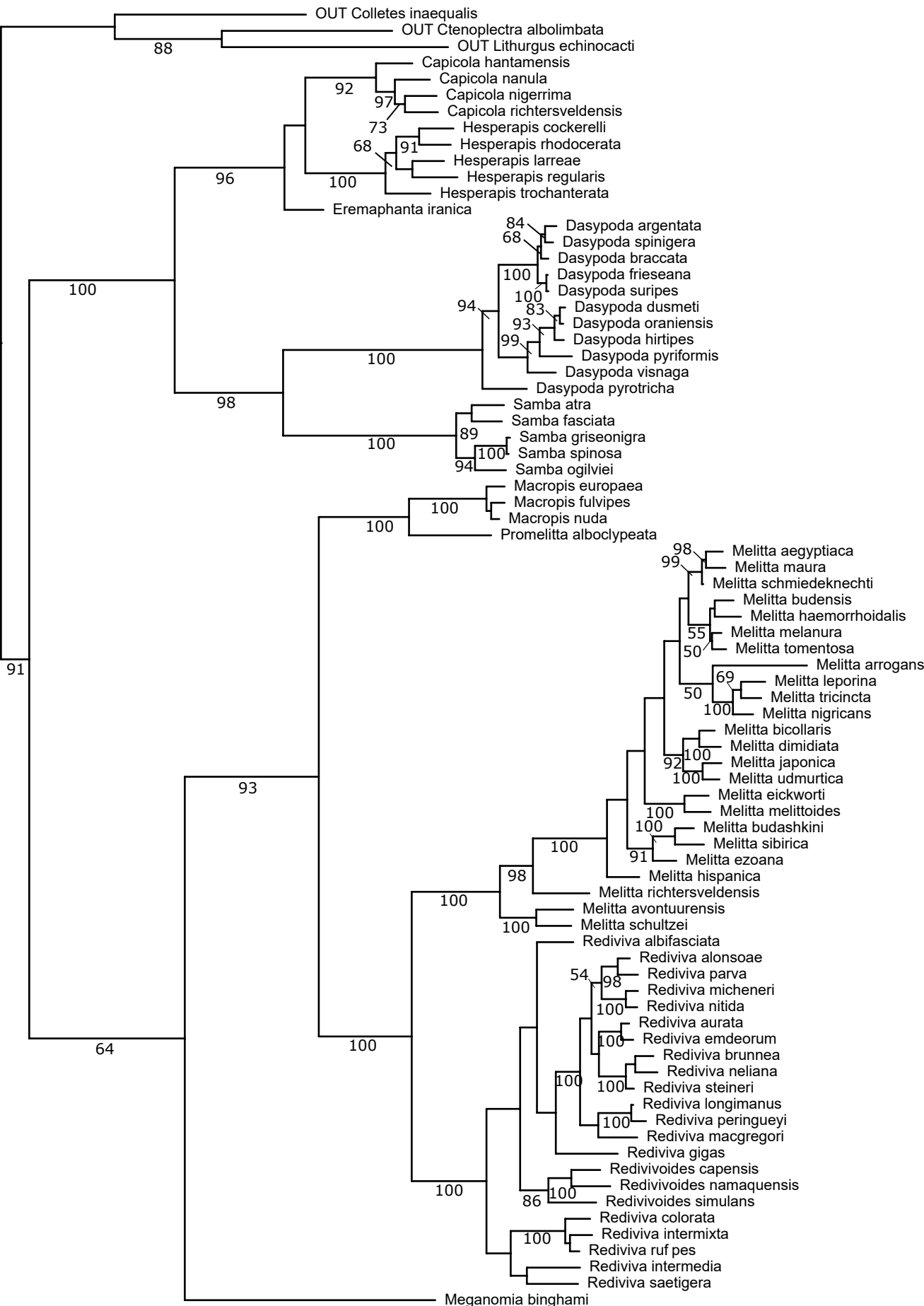

Supplement: S2 Fig — Numbers below nodes are bootstrap support values. (PDF) [file pone.0217839.s010.pdf]

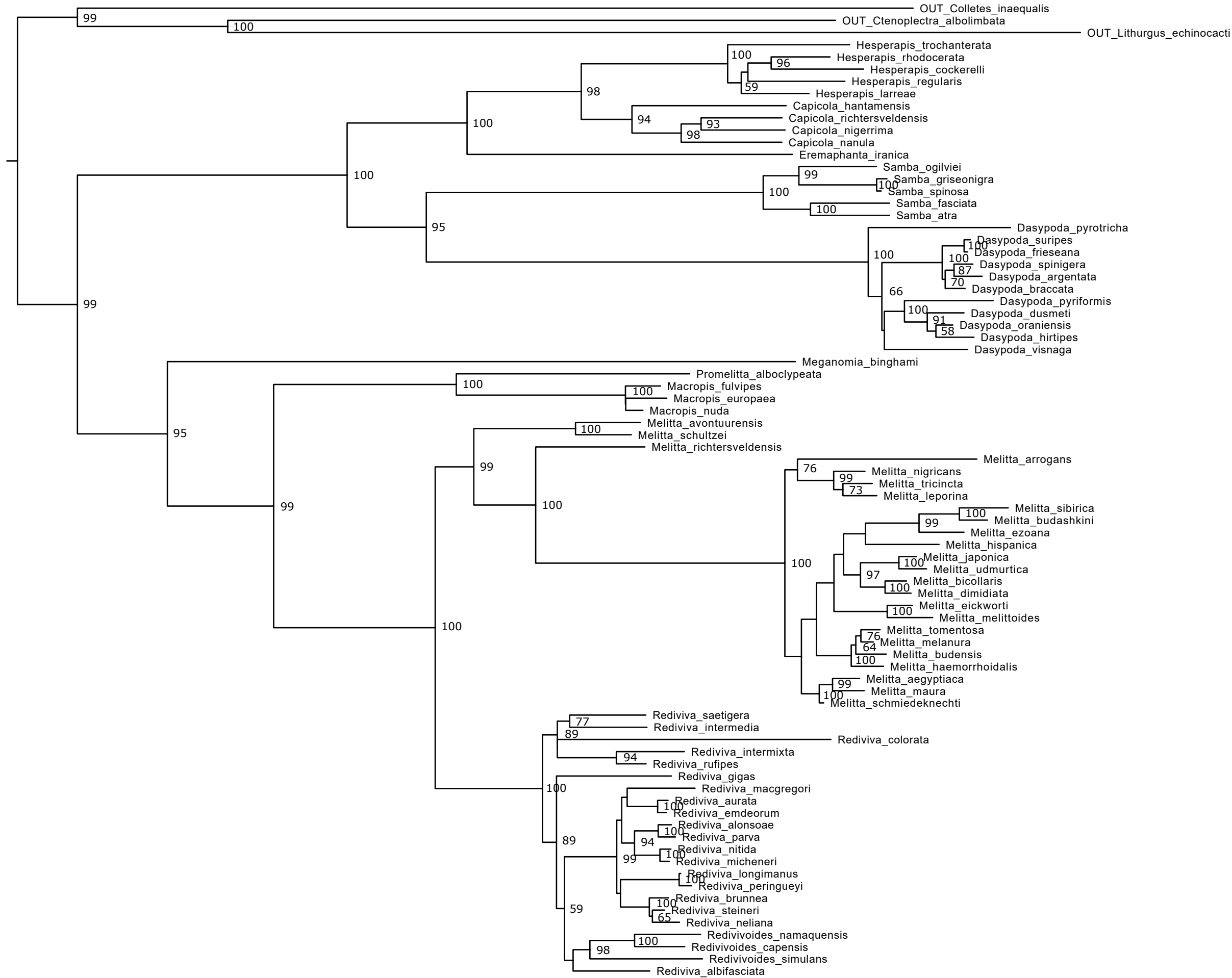

0.03

Supplement: S3 Fig — Numbers below nodes are bootstrap support values. (PDF) [file pone.0217839.s011.pdf]
