# Supplementary material for: The allometry of proboscis length in Melittidae (Hymenoptera: Apoidae) and an estimate of their foraging distance using museum collections
Source: PLoS One. 2019 Jun 7;14(6):e0217839. doi: 10.1371/journal.pone.0217839 (PMC6555519; doi:10.1371/journal.pone.0217839)
Supplement: S1 Methods & Results — (DOCX) [file pone.0217839.s012.docx]

**SI Methods & Results: construction of an updated phylogenetic hypotheses of the Melittidae**

**Methods**

We downloaded data from Genbank for 77 species (S6 Table). These data were sourced primarily from the following four papers: Michez et al. (2009; 2010); Dellicour et al. (2014); and Kahnt et al. (2017) – with Genbank accession numbers provided in S6 Table. The dataset comprised eight genes (7 nuclear, 1 mitochondrial): 28S ribosomal gene (1088 bp); EF1α (F2 copy; 1071 bp); NaK (1465 bp); Opsin (632 bp); RNAp (888 bp); WgL (462 bp); CAD (1152 bp); mitochondrial COI (741 bp). All genes, except 28S, code for proteins, and only the exons were included in the alignment (S7 Table). Alignments were performed computationally using MAFFT (Katoh et al. 2009) and checked by eye. Alignment was straight-forward for all loci with the exception of 28S, from which positions 102-180 (79 bp), 297-348 (52 bp), 411-466 (56 bp), 492-566 (75 bp), 632-700 (69 bp), 791-805 (15 bp), 898-917 (20 bp), 1312-1317 (6 bp), 1331-1343 (13 bp) were excluded. The final alignment was 7,499 bp for 80 species that included three outgroups: *Colletes inaequalis*, *Ctenoplectra albolimbata*, and *Lithurgus echinocacti* (S6 Table).

To construct phylogenetic hypotheses we made use of two optimality criteria, parsimony and maximum likelihood. The parsimony analyses were implemented using PAUP* (Swofford 2002) with a heuristic search and 1000 random addition replicates with TBR branch-swapping. Node support was assessed using 100 bootstrap replicates with 5 random addition replicates per bootstrap replicate. Maximum likelihood analyses were performed using RAXML v8.2.10 (Stamatakis 2014) on the CIPRES portal (Miller et al. 2010) using a GTR+I model of nucleotide substitution and 10 data partitions (each nuclear locus, and for COI a different partition for each codon). Node support was determined via rapid bootstrapping, with RAXML halting bootstrapping automatically in accordance with the MRE-based criterion (Stamatakis 2014). The dating of nodes using external calibration points was not performed, rather branch-lengths are scaled relative to one another.

**Results**

The data matric of 80 species and 7,499 characters comprised 2022 parsimony informative characters, 557 variable but parsimony uninformative characters, and 4920 constant characters. The maximum parsimony analyses using a heuristic search in PAUP* recovered 48 trees of length 8617. One of these trees is presented as S2 Fig. The maximum likelihood analyses recovered a single tree of length – ln 51437.698804 (S3 Fig). Rapid bootstrapping halted after 504 replicates.

Dellicour, S., Lecocq, T., Kuhlmann, M., Mardulyn, P., and Michez, D. (2014). Molecular phylogeny, biogeography, and host plant shifts in the bee genus *Melitta* (Hymenoptera: *Anthophila*). Molecular Phylogenetics and Evolution. 70: 412-419.

Kahnt, B., Montgomery, G.A., Murray, E., Kuhlmann, M., Pauw, A., Michez, D., Paxton, R.J. and Danforth, B.N. (2017). Playing with extremes: origins and evolution of exaggerated female forelegs in South African *Rediviva* bees. Molecular Phylogenetics and Evolution. 115: 95-105.

Katoh, K., Asimenos, G., Toh, H., 2009. Multiple alignment of DNA sequences with MAFFT. Methods in Molecular Biology. 537: 39–64.

Michez, D., Patiny, S. and Danforth, B.N. (2009). Phylogeny of the bee family Melittidae (Hymenoptera: Anthophila) based on combined molecular and morphological data. Systematic Entomology. 34: 574-597.

Michez, D., Eardley, C., Kuhlmann, M., Timmermann, K. and Patiny, S. (2010). The bee genera Haplomelitta and Samba (Hymenoptera: Anthophila: Melittidae): phylogeny, biogeography and host plants. Invertebrate Systematics. 24: 327-347.

Miller, M.A., Pfeiffer, W., Schwartz, T., 2010. Creating the CIPRES Science Gateway for inference of large phylogenetic trees. In: Proceedings of the Gateway Computing Environments Workshop (GCE), New Orleans. pp 1–8.

Stamatakis, A. (2014) RAxML Version 8: A tool for Phylogenetic Analysis and Post-Analysis of Large Phylogenies. Bioinformatics 10.1093/bioinformatics/btu033.

Swofford, D.L., 2002. PAUP*10b: Phylogenetic Analysis Using Parsimony (*And other Methods). Sinauer Associates, Sutherland, Massachusetts.
